# Natural zeolite for heavy metal, ammonia removal, and physiological responses in European sea bass (*Dicentrarchus labrax*) juveniles tanks with different densities

**DOI:** 10.1371/journal.pone.0297844

**Published:** 2024-04-05

**Authors:** Ghada R. Sallam, Hadir A. Aly, Ayman M. Lotfy, Mohamed M. Abdel-Rahim, Walied M. Fayed, Islam I. Teiba, Kumbukani Mzengereza, Mavuto Tembo, Wales Singini, Yusuf Jibril Habib, Akram Ismael Shehata

**Affiliations:** 1 Aquaculture Division, Fish Rearing Lab., National Institute of Oceanography and Fisheries (NIOF), Alexandria, Egypt; 2 Department of Animal and Fish Production, Faculty of Agriculture (Saba Basha), Alexandria University, Alexandria, Egypt; 3 Faculty of Agriculture, Tanta University, Tanta, Egypt; 4 Department of Fisheries and Aquatic Science, Mzuzu University, Mzuzu, Malawi; 5 African Center of Excellence in Neglected and Underutilized Biodiversity, Mzuzu University, Mzuzu, Malawi; 6 Department of Medical Analysis, Tishk International University-Erbil, Kurdistan Region, Iraq; Tanta University Faculty of Agriculture, EGYPT

## Abstract

The present study aims to investigate the influence of zeolite usage and stocking densities on various parameters, including ammonia removal from water, accumulation of heavy metals in fish organs, water quality, growth performance, feed efficiency, muscle composition, as well as hematological and biochemical parameters in European seabass (*Dicentrarchus labrax*) over a 90-day duration. A total of 2400 *D*. *labrax* with an initial weight of 9.83 ± 2.02 g and initial length of 9.37 ± 0.32 cm were distributed among 24 tanks. The research involved six distinct treatment groups, with two different zeolite levels (0 and 15 ppt) and three stocking density levels (50, 100, and 150 fish/m^3^), each replicated four times. The results of the research demonstrate a statistically significant improvement (*p* < 0.05) in water quality measures with the introduction of zeolite. The successful implementation of this amendment mitigated the adverse effects of fish density on water quality parameters. Higher stocking density negatively impacted European sea bass growth, feed utilization, and hemato-biochemical indicators. Zeolite use effectively alleviated these adverse effects, particularly on performance, feed utilization, hematological, and biochemical parameters. The study’s results indicate that the utilization of zeolite has shown to be efficacious in mitigating the accumulation of heavy metals in both water and fish organs, while concurrently augmenting fish attributes. However, the increase in density led to a significant decrease in the accumulation of heavy metals in both water and fish organs. The present study highlights the capacity of natural zeolites to mitigate the negative consequences associated with water quality concerns. The efficiency of these zeolites in limiting the accessibility of heavy metals in polluted water is shown, hence minimizing their accumulation in fish organs. In addition, the improvement of fish performance has the capacity to have a beneficial influence on both the well-being and efficiency of fish in aquaculture. Additional research is essential to fully understand the complex molecular pathways involved in utilizing natural zeolite under different fish densities.

## 1. Introduction

Feeding the world’s expanding population, which is expected to reach 9.6 billion by 2050, poses a significant problem due to a lack of natural resources required for food production, such as land and water. Because of limited land and water resources, aquaculture’s sustainability will most likely depend on improving production settings, boosting productivity, upgrading aquaculture technology, and lowering production costs. In many nations, aquaculture has emerged as an excellent food production alternative [1, 2]. The aforementioned industry has seen rapid growth because of its pivotal role in addressing the increasing need for animal protein in the human diet [3]. Sustainable aquaculture might help accomplish FAO Sustainable Development Goals to reduce poverty, hunger, food security, nutrition, and sustainable agriculture [1, 4]. Because of subsequent industrial development, the excessively wasteful use of agricultural pesticides in recent and previous decades, and the disregard for safe disposal methods for industrial waste, most of the water resources used for fish farming have been contaminated, particularly by heavy metal pollutants [5].

The use of all accessible water sources, even dubious drinking water, is a novel aquaculture approach. Thus, many aquaculture industry chose groundwater as their primary water supply [6]. Aquaculture in the desert using subsurface brackish or saltwater is a potential option. Desert aquaculture may be a sustainable production industry, particularly in areas where plant cultivation is difficult [7–9]. However, people who use deep saltwater have a lot of problems, like water that is too hard, has too much ammonia, and not enough air [10–12]. That being said, this water supply is susceptible to rapid degradation based on pond biomass or stocking density. Intensified manufacturing techniques are now needed to fulfill the growing demand brought on by population growth. The provided area for aquaculture growth, the scarcity of freshwater, and the restrictive wastewater rules are the main hindrances to long-lasting conventional systems [13, 14]. In order to establish a sustainable sector for dessert aquaculture, several studies have examined ways to improve subterranean seawater quality via the use of different technologies, including activated carbon [15, 16], probiotic and biofloc technology system [17, 18], biological and sand filters [19–21], and zeolites [22–25]. Fish are near the top of the marine food chain, so heavy metals can usually get into their bodies from food, water, and sediments [26–28].

The presence of hazardous heavy metals in fish may negate their good benefits; various deleterious effects of heavy metals on human health have long been recognised [29]. This could include very bad threats like kidney failure, liver damage, heart disease, and even death [30, 31]. Also, stocking density (SD) is an important component influencing the physical and biological properties of fish. Besides that, SD might affect the quality of the water because a high SD can cause a lot of ammonia and other bad things to be in it. Ammonia is the second-most important water quality factor that affects fish performance, especially in systems that use a lot of water [32].

Heavy metals (Zn, Cr, Pb, Cd, Cu, Mn, Fe, etc.) are also a big problem for the environment because they are found in the ocean, groundwater, and wastewater [33], which in turn adversely affects aquaculture projects. As industry trash, a lot of dangerous heavy metals have been dumped into the environment, polluting the land and water badly. Cadmium, copper, nickel, lead, and zinc are some of the most common metals that build up in living organisms and cause a wide range of illnesses and disorders [34]. Additionally, they are frequent pollutants in groundwater in military and industrial locations. There are many methods for extracting dissolved heavy metals, including electrodialysis, sorption, phytoextraction, precipitation, ultrafiltration, and reverse osmosis [35–37]. Recent discourse has cantered on the possibility of utilizing other inexpensive products as sorbents for heavy metal removal. Nevertheless, excessive levels of heavy metals and ammonia that cause injury can be remedied in a variety of ways. Mechanical solutions and chemical solutions are two such economically prohibitively expensive methods. The economy and technology are both receptive to alternative approaches. The addition of natural zeolite to aquaculture water is one of the most promising methods for purifying water [12, 25, 38, 39].

Zeolites are hydrated aluminosilicate minerals that occur naturally. They belong to the mineral class known as "tectosilicates." The most prevalent natural zeolites are generated through glass alteration. Many authors have explored the sorption capacity of natural zeolite (clinoptilolite) for inorganic cations [40, 41]. More research is showing that natural zeolite has a lot of potential to be one of the best and most cost-effective ways to remove ammonia and some heavy metals from water. This is because it has special molecular sieve, sorption, and ion exchange properties [33]. Because zeolite exchangeable ions are largely harmless (sodium, calcium, and potassium ions), they are especially well suited for removing unwanted heavy metal ions from industrial effluent waters. Clinoptilolite is the most prevalent natural zeolite, and much study has been done to characterize its chemical, surface, and sorption characteristics [42]. It is possible to get rid of some heavy metals from rainwater with zeolites. When it comes to sorption processes, clinoptilolite samples from different areas behave in different ways. This study looked at how well a natural zeolite called Clinoptillolite from Western Anatolia can soak up some heavy metal cations that are in solution. So, the main goal of this work is to use natural zeolite to change the damaging aquaculture waste water from high SD ponds where European sea bass are raised and that rely on underground salt water.

## 2. Material and method

### 2.1. Ethical approval

All guidelines for rearing and using fish were followed and approved according to the Institutional Animal Care and Use Committee (IACUC) at the National Institute of Oceanography and Fisheries, Egypt, which licensed ethical permission (Approval No. NIOF. AQ1. F. 21. R. 005). Furthermore, all research procedures adhered to the ARRIVE guidelines v2.0 [43], ensuring the research protocol aligns with established ethical standards and safeguards the well-being of the fish subjects.

### 2.2 Experimental location and facilities

Ethics rules from the National Institute of Oceanography and Fisheries, Egypt, were followed during the trial. Two thousand four hundred healthy European sea bass fingerlings were bought from the Marine Finfish Hatchery, K21, which is part of the General Authority for Fish Resources Development (GAFRD) and the Ministry of Agriculture and Land Reclamation in West Alexandria, Egypt. They were then kept at the El-Mothalas Fish Rearing Facility at the El-Max Station for Applied Research, which is part of the National Institute of Oceanography and Fisheries (NIOF) in Alexandria, Egypt. The Mediterranean Sea is 1.2 kilometers away from this spot. Deep wells (about 100 m deep) are the only places that can provide saltwater with a salinity of 32<. Unfortunately, tests on water samples showed that the levels of ammonia and heavy metals were a little higher than what is suggested for marine fish hatcheries [44]. Certain heavy metals include: Iron (99.3 μg/l), cobalt (50 μg/l), copper (5.3 μg/l), manganese (85.2 μg/l), zinc (6.5 μg/l), cadmium (40 μg/l), chrome (66 μg/l), nickel (70,113 μg/l), and lead (28 μg/l) [45].

### 2.3 Experimental design and fish

After acclimating the fish for fifteen days, 2400 sea bass fingerlings (average initial weight: 9.83 ± 2.02 g and initial length of 9.37 ± 0.32 cm) were relocated to 24 experimental concrete containers (3.75 m^3^). Three stocking densities (50, 100, and 150 fish/m^3^) were tested with 15ppt zeolite concentration and control treatments (same densities without zeolite). The selection of the zeolite concentration was determined by findings from a prior study of Mansour et al., [25]. Every week, the zeolite sacs were taken out, cleaned with fresh water, let to air dry, and then reused for a maximum of four weeks before being swapped out for new ones. The natural zeolite (clinoptilolite, denoted as Z) employed in this experiment was sourced from Yemen (http://alixzeolite.com/en/). Its physical and chemical characteristics have been detailed in previous studies [12, 25, 38, 39].

### 2.4 Feed formulation and feeding protocol

The fish were fed with a home-formulated pelleted diet. In line with recommendations from a prior experiment, considerable effort was invested in meticulously formulating the experimental diet to ensure it comprised 38% crude protein and 12% crude lipid, aligning with the specific nutritional requirements of the target species (*D*. *labrax*) [46]. **Table 1** displays the formulation and approximate composition (%, on a dry matter (DM) basis) of the diet. Fish were fed to satiety four times per day for ninety days.

**Table 1:** Composition and proximate analysis of diet used during the study.

Ingredients	(%)	Proximate Analysis	(%)
Fish meal	36.00	Dry mater	91.66
Wheat flour	11.70	Crude protein (N × 6.25)	37.81
Wheat bran	11.20	Crude lipid	12.6
Soybean meal	18.00	Crude fiber	2.4
Yellow corn	14.80	Carbohydrate (NFE)^3^	30.75
Fish oil	4.10	Ash	8.1
CMC^2^	3.00	Gross energy k.cal/100g	465.66
Vit.&Min.Mix^1^	0.80		
Ascorbi acid	0.40		

^1^Vitamin and mineral mixture / kg premix: Vitamin A, 4.8 million IU; D3, 0.8 million IU; E, 4 g; K, 0.8 g; B1 0.4 g; riboflavin, 1.6 g; B6 0.6 g; B12 4 mg; Pantothinic acid, 4 g; Nicotinic acid, 8 g; Folic acid, 0.4 g; Biotin, 20 mg; Choline chloride, 200 g; cu, 4 g; I, 0.4 g; Iron, 12 g; Mn, 22 g; Zn, 22 g; Selenium, 0.4 g.

^2^ CMC, Carboxymethylcellulose.

^3^ NFE, Nitrogen free extract.

### 2.5 Sample collection and analytical procedures

#### 2.5.1 Water analysis

The water quality parameters were monitored, including temperature (~ 26–28°C), pH, dissolved oxygen (DO, mg/L), salinity (~ 28–29 ppt), and total ammonia nitrogen (TAN, ppm) on a daily basis for all treatment groups. A portable PH meter (PH-8424) (HANNA Instrument) was used to test both pH and temperature. The measurement of dissolved oxygen was done using the HI-9142 (HANNA Instrument). The YSI Eco Sense EC300 Conductivity/Salinity meter was used to monitor salinity. Utilizing the YSI Professional Plus and YSI 9300 photometers, total ammonia nitrogen (TAN) was tracked. The U.S. Environmental Protection Agency determined the concentration of un-ionized ammonia-N as a percentage of TAN. (www.epa.gov/oeca/disclaimer.html).

#### 2.5.2 Determination of metal concentrations and quality assurance

Copper (Cu), lead (Pb), cadmium (Cd), iron (Fe), and zinc (Zn) were analyzed in water (μg/L) and fish organs (mg/g wet weight,wt). Heavy metal levels were measured by sampling each tank’s water three times a week. Using Atomic Absorption Spectrophotometer (AAS), samples were examined. Water examination of heavy metals followed Shkinev et al., [47], whereas fish samples were analyzed using Atta et al., [48]. Fish samples were washed with distilled water and scales were removed. The fish were dissected and the muscle tissue, liver, and gills separated using stainless steel instruments and digested as stated [48, 49]. The procedure included breaking down 1 gram of ingredients using a 1:1 mixture of perchloric acid, nitric acid, and sulfuric acid. The mixture was then heated at 200° C for 30 minutes. Once the digest had cooled to room temperature, it was mixed with pure water to make 50 ml. It was then tested for Cu, Zn, Pb, and Cd using an atomic absorption spectrophotometer (AAS type Agilent AA55) at various wavelengths. The same steps were used to make analytical blanks. The answer was mg/kg of wet weight.

### 2.6 Growth performance, survival and feed utilization

At day 90 of the experiment, the mean body weight (FBW, g) of the experimental treatments was obtained by randomly weighing 15 fish from each tank and dividing the total weight by the fish count. Furthermore, the condition factor (KF) of both treated and untreated *D*. *labrax* juveniles was computed. The data were then used to determine if zeolite improved the growth of *D*. *labrax* juveniles. According to Cho and Kaushik [50] and Castell and Tiews [51], the growth performance, feed utilization, survival rate and hepatosomatic index of fish juveniles were determined using the following equations.

**Weight gain (WG, g)** = final body weight (g)-initial body weight (g)
**Average daily gain (ADG, g/fish/d)** = (Final Weight—Initial Weight) / Number of Days
**Specific growth rate (SGR, %/d)** = 100 × [(ln final body weight (g)-ln initial body weight (g))/ duration of rearing (day)], where ln is the natural logarithm.
**Survival rate (SR,%)** = 100 × (initial number of fish/final number of fish).
**Hepatosomatic Index (HSI)** = (Liver Weight / Total Body Weight) × 100
**Feed intake (FI, g/fish)** = Total feed supplied—Remaining feed
**Feed conversion rate (FCR)** = feed intake (g)/weight gain (g).
**Protein efficiency rate (PER)** = net weight gained (g)/protein intake amount (g).
**Condition factor (KF)** = 100W/L^3^, where W = fish weight; L = fish length.

### 2.7 Feed and fish proximate chemical analyses

Prior to the commencement of the experiment, a random sample of the feed and the fish under investigation (approximately fifty fish) were retained in order to ascertain their initial body chemistry composition. Following the conclusion of the experiment, 15 fish from each treatment were collected as samples in order to identify any nearby contaminants. Samples of the trial feed and fish underwent thorough chemical analysis following AOAC [52] guidelines to assess their moisture, crude protein, crude lipid, crude fiber, and ash contents in the diet. Additionally, protein, lipid, and ash concentrations were analyzed in the whole body of European sea bass as part of the comprehensive evaluation.

### 2.8 Blood sample: Hematology

The fish underwent anesthesia using 0.3 ml/l of clove oil prior to blood collection. Subsequently, blood samples were drawn from three fish per replicate and twelve fish per treatment via the caudal vein and collected in plastic tubes. Using a 1 ml syringe, blood samples were obtained at the end of the experiment by puncturing the caudal vein. To examine the hematological profile, samples were obtained in tiny plastic tubes containing ethylene diamine tetra acetic acid (EDTA), an anti-coagulating agent. Using Drabkin’s solution and the cyanmethemoglobin technique, the evaluation of hemoglobin concentration (Hgb) was calculated [53].

#### 2.8.1 Serum constituents

Blood serum was collected without EDTA, allowed to clot at room temperature, and centrifuged at 3000 rpm for 20 minutes. The serum samples were kept at—20°C until analysis. The concentration of glucose was assessed through colorimetric methods as outlined in the procedure detailed by Trinder [54]. Cholesterol (mg/dl) [55], aspartate aminotransferase activity (AST, EC. 2.6.1.1), alanine aminotransferase activity (ALT, EC. 2.6.1.2) [56], and serum urea level [57] were measured using enzymatic methods. El-Nasr Pharmaceutical Chemicals Co. (Egypt) kits were used to calorimetrically identify serum total protein (g/dl) according to the manufacturer’s instructions and the following references: total protein [58] and albumin content [59]. After subtracting albumin (A) from the total protein level, the A/G ratio was computed to determine globulin (G).

### 2.9 Statistical analysis

To examine differences among the treatments, all data from the experimental trial were statistically analyzed using R, as described by Assaad et al., [60]. The data were analyzed using two-way analysis of variance (ANOVA) with post hoc analysis and the Tukey’s test, with *P* < 0.05 set as the criterion of significance. The ANOVA method was used to calculate the influence of the zeolite treatment on the survival rate, water quality, growth, and physiological parameters of juvenile European seabass. The data are shown in a tabular format (meaning standard errors).

## 3. Results

### 3.1 Heavy metal concentrations in water samples

The mean levels of certain heavy metals (cadmium, copper, zinc, iron, and lead) in water at varying densities for the control and zeolite groups **(Table 2)**. The results presented in demonstrate the concentrations of heavy metals (Cd, Cu, Zn, Fe, and Pb) in water under different treatments, varying in fish density (50, 100, and 150 fish) and the inclusion of zeolite. The values, expressed in micrograms per liter (μg/L) and presented as means ± standard error (SE), reveal significant variations among treatments. For cadmium (Cd), a clear trend of decreasing concentrations is observed with increasing fish density, further accentuated by the addition of zeolite. Copper (Cu), zinc (Zn), iron (Fe), and lead (Pb) also exhibit notable changes in concentration across treatments. Statistical analysis (*P-values*) underscores the significance of both fish density and zeolite application in influencing heavy metal concentrations, with interactions between these factors also noted. These findings highlight the potential of fish density management and zeolite supplementation as effective strategies for controlling heavy metal levels in aquatic environments.

**Table 2:** Heavy metals concentration (μg /L) in water of different treatments (Means± SE).

Variable	Control			Zeolite			*P*-value		
	Density 50	Density 100	Density 150	Density 50	Density 100	Density 150	Treat	Density	Interac
Cd	20.25 ± 0.63^a^	17.5 ± 0.65^ab^	14.75 ± 0.48^bc^	13.5 ± 0.65^c^	10.5 ± 0.65^d^	5.5 ± 0.65^e^	<0.001	<0.001	0.112
Cu	2070 ± 4.09^a^	2067 ± 2.78^a^	2062 ± 1.66^a^	1741 ± 0.85^b^	1734 ± 0.85^b^	1519 ± 0.63^c^	<0.001	<0.001	<0.001
Zn	5193 ± 21.44^a^	5172 ± 20.78^a^	5088 ± 2.78^b^	4139 ± 8.69^c^	2090 ± 0.75^d^	1468 ± 1.32^e^	<0.001	<0.001	<0.001
Fe	332.8 ± 5.68^a^	327.2 ± 2.78^a^	321.5 ± 0.29^a^	175.8 ± 0.25^b^	121.5 ± 0.29^c^	102.8 ± 0.25^d^	<0.001	<0.001	<0.001
Pb	12.5 ± 0.29^a^	11.5 ± 0.29^a^	11.75 ± 0.25^a^	7.25 ± 0.25^b^	4.5 ± 0.29^c^	3.25 ± 0.48^c^	<0.001	<0.001	<0.001

Density, (fish/m^3^)

### 3.2 Ammonia removal rate, source and ammonia removal rate, control heavy metal concentration

**Table 3** presents the ammonia removal rates (ARRS and ARRC, expressed as percentages of the source and control, respectively) for various heavy metals under different treatments, including those involving fish densities treated both with and without zeolite. The results, presented as means with standard errors, highlight substantial variations in ammonia removal efficiency. Zeolite application at different fish densities significantly enhances ammonia removal rates compared to the control. Notably, the removal rates exhibit a clear dose-dependent relationship, with higher fish densities demonstrating superior ammonia removal. The zeolite-treated groups consistently outperform the control, as evidenced by the significantly higher RRS percentages for Cd, Cu, Zn, Fe, and Pb. Additionally, the ARRC percentages underscore the effectiveness of zeolite in augmenting ammonia removal compared to the control, with significant differences observed across all metals. The interaction effects between treatment and fish density further emphasize the nuanced interplay influencing ammonia removal rates in this experimental setup. Overall, the results underscore the potential of zeolite treatment in enhancing ammonia removal efficiency in aquatic environments.

**Table 3:** Heavy metals concentration in ammonia removal rate; as % of the source (ARRS) and ammonia removal rate; as % of the control (ARRC) of different treatments (Means± SE).

Variable	Control			Zeolite			*P*-value		
	Density 50	Density 100	Density 150	Density 50	Density 100	Density 150	Treat	Density	Interac
**RRS%**									
Cd	10.86 ± 2.06^e^	22.66 ± 4.46^de^	35.12 ± 0.68^cd^	40.26 ± 4.23^bc^	53.4 ± 4.27^b^	75.57 ± 3.28^a^	<0.001	<0.001	0.244
Cu	0.35 ± 0.1^c^	0.50 ± 0.17^c^	0.73 ± 0.24^c^	16.22 ± 0.21^b^	16.53 ± 0.27^b^	26.88 ± 0.20^a^	<0.001	<0.001	<0.001
Zn	4.28 ± 1.41^d^	4.68 ± 1.04^d^	6.21 ± 1.33^d^	23.71 ± 1.09^c^	61.48 ± 0.53^b^	72.95 ± 0.38^a^	<0.001	<0.001	<0.001
Fe	5.9 ± 1.85^c^	7.38 ± 2.43^c^	8.95 ± 3.02^c^	50.22 ± 1.7^b^	65.59 ± 1.18^a^	70.89 ± 1.03^a^	<0.001	<0.001	0.001
Pb	9.07 ± 1.75^c^	16.35 ± 1.71^c^	14.42 ± 2.80^c^	47.25 ± 1.72^b^	67.31 ± 1.80^a^	76.24 ± 3.74^a^	<0.001	<0.001	<0.001
**RRC%**									
Cd	0 ± 0^c^	0 ± 0^c^	0 ± 0^c^	72.06 ± 6.542^a^	58.25 ± 5.3^ab^	53.25 ± 2.37^b^	<0.001	0.044	0.044
Cu	0 ± 0	0 ± 0	0 ± 0	97.87 ± 0.5678^a^	97 ± 0.96^a^	97.3 ± 0.89^a^	<0.001	0.75	0.75
Zn	0 ± 0^b^	0 ± 0^b^	0 ± 0^b^	82.59 ± 5.504^a^	92.42 ± 1.6^a^	91.51 ± 1.78^a^	<0.001	0.114	0.114
Fe	0 ± 0^b^	0 ± 0^b^	0 ± 0^b^	88.56 ± 3.295^a^	88.93 ± 3.5^a^	87.56 ± 4.12^a^	<0.001	0.964	0.964
Pb	0 ± 0^b^	0 ± 0^b^	0 ± 0^b^	80.95 ± 3.224^a^	75.83 ± 1.9^a^	81.24 ± 3.38^a^	<0.001	0.359	0.359

Density, (fish/m^3^)

### 3.3 The determination of heavy metal concentrations in European sea bass tissues

Heavy metals like Fe, Cu, Zn, Cd, and Pb (mg/g wet wt.) were found in sea bass fingerlings’ gills, liver, and muscles at different concentrations, with and without zeolite treatment (**Table 4**). All metal accumulation patterns varied significantly (*p* < 0.001) among treatments and fish organs. **Table 4** reveals that all fish had the least amount of metals (Fe, Cu, and Zn) in their muscles, whereas almost all fish had the most Fe, Cu, and Zn in their liver. Tukey’s test indicated metal variations, such as the highest levels of Fe, Cu, and Zn in the liver in different treatments, whereas muscles had the lowest concentration of all metals. When investigating how metals fluctuate in water based on the density of the fish, **Table 4** indicates that adding zeolite significantly decreased heavy metal accumulation (*p* < 0.001) in all organs of sea bass of all densities. Furthermore, higher fish density is linked to lower heavy metal accumulations in all organs of sea bass.

**Table 4:** Means± SE of heavy metals concentration (mg/g wet weight) in different organs of European sea bass in different treatments.

Variable	Control			Zeolite			*P*-value		
	Density 50	Density 100	Density 150	Density 50	Density 100	Density 150	Treat	Density	Interac
**Liver**									
Fe	677.5 ± 13.22^a^	436.1 ± 6.21^b^	142.8 ± 5.66^c^	444.1 ± 27.5^b^	138.6 ± 6.61^c^	120.3 ± 3.6^c^	<0.001	<0.001	<0.001
Cu	7.44 ± 0.23^a^	5.17 ± 0.08^b^	5.16 ± 0.14^b^	5.19 ± 0.39^b^	3.17 ± 0.08^c^	2.28 ± 0.13^c^	<0.001	<0.001	0.117
Zn	110.2 ± 3.93^a^	75.27 ± 2.71^b^	55.3 ± 2.18^c^	82.68 ± 3.39^b^	43.18 ± 3.56^cd^	32.24 ± 1.49^d^	<0.001	<0.001	0.344
Pb	0.3 ± 0.01^a^	0.23 ± 0.03^ab^	0.21 ± 0.01^b^	0.16 ± 0.02^c^	0.13 ± 0.01^c^	0.12 ± 0.00^c^	<0.001	0.003	0.302
Cd	0.6 ± 0.13^a^	0.76 ± 0.1^a^	0.61 ± 0.03^a^	0.49 ± 0.02^ab^	0.25 ± 0.03^bc^	0.15 ± 0.02^c^	<0.001	0.083	0.023
**Gills**									
Fe	139.5 ± 6.53^a^	112.7 ± 0.91^b^	84.14 ± 3.063^c^	98.85 ± 4.11^bc^	84.76 ± 2.81^c^	57.08 ± 2.6^d^	<0.001	<0.001	0.156
Cu	10.16 ± 0.56^a^	6.37 ± 0.13^b^	4.48 ± 0.21^c^	2.74 ± 0.05^d^	1.57 ± 0.15^e^	1.09 ± 0.08^e^	<0.001	<0.001	<0.001
Zn	78.7 ± 3.45^a^	50.04 ± 0.10^b^	32.9 ± 1.12^c^	54.07 ± 2.90^b^	34.73 ± 2.66^c^	21.73 ± 1.33^d^	<0.001	<0.001	0.023
Pb	5.46 ± 0.34	3.87± 0.27	2.92 ± 0.08	1.64 ± 0.13	1.16 ± 0.04	22.78 ± 21.74	0.548	0.455	0.344
Cd	1.26 ± 0.04^a^	0.82 ± 0.1^b^	0.67 ± 0.02^bc^	0.55 ± 0.02^cd^	0.34 ± 0.03^de^	0.24 ± 0.02^e^	<0.001	<0.001	0.02
**Muscles**									
Fe	56.77 ± 2.00^a^	41.94 ± 1.03^b^	32.33 ± 1.15^c^	40.77 ± 1.25^b^	31.5 ± 1.25^c^	22.2 ± 0.7^d^	<0.001	<0.001	0.061
Cu	6.15 ± 0.14^a^	1.95 ± 0.09^c^	1.012 ± 0.03^d^	2.89 ± 0.10^b^	1 ± 0.07^d^	0.69 ± 0.04^d^	<0.001	<0.001	<0.001
Zn	54.69 ± 2.3^a^	51.16 ± 1.61^ab^	43.4 ± 1.68^bc^	47.28 ± 3.35^ab^	35.11 ± 1.77^cd^	27.28 ± 1.58^d^	<0.001	<0.001	0.092
Pb	0.53 ± 0.04^a^	0.27 ± 0.03^b^	0.18 ± 0.02^bc^	0.17 ± 0.02^bc^	0.13 ± 0.01^c^	0.11 ± 0.00^c^	<0.001	<0.001	<0.001
Cd	0.09 ± 0.01^a^	0.06 ± 0.01^ab^	0.07 ± 0.02^ab^	0.06 ± 0.01^ab^	0.03 ± 0.00^b^	0.02 ± 0.004^b^	0.001	0.005	0.567

Density, (fish/m^3^)

### 3.4 Efficacy of ammonia removal and water quality

The result of the water quality parameters of European seabass when treated with zeolite as removal of ammonia concentration was shown in **Table 5**. The data demonstrated that when density increased, dissolved oxygen (DO, mg/L), pH, and nitrate (NO_3_, mg/L) levels declined considerably (*p* < 0.05). However, ionized ammonium (NH_4_, mg/L), unionized ammonia (NH_3_, mg/L), and nitrite (NO_2_, mg/L) rise. The results also showed that the zeolite treatment significantly improved (*p* < 0.05) the water quality parameters by lowering NH_4_, NH_3_, and NO_2_ levels compared to the control treatments. The addition of zeolite resulted in significant DO, pH, and ammonia readings. In addition, for all water quality metrics, there was a substantial relationship between stocking density (SD) and zeolite treatment. The results revealed that pH and DO increased considerably (*p* < 0.05) from 6.7 and 4.8 with high density in the control treatment to 7.95 and 6.75 with low density in the zeolite treatment. Throughout the research period (**Table 5**), the overall values of ionized ammonium (NH_4_), unionized ammonia (NH_3_), and nitrite (NO_2_) were substantially different (*p* < 0.05) among the tested treatments. The addition of natural zeolite had an inverse connection with all ammonia readings. It was found that stocking densities greatly (*p* < 0.05) raised ammonia levels. The tested zeolite did better than the control treatments when it came to removing ammonia from the source water. The zeolite treatment with a low stocking density had the best results, with a 63% removal rate. With decreasing stocking density, the value of the ammonia removal rate as a proportion of the source water rises.

**Table 5:** Water quality parameters of European seabass treated with natural zeolite as removal of stressful ammonia concentrations.

Variable	Sours	Control			Zeolite			*P*-value		
		Density 50	Density 100	Density 150	Density 50	Density 100	Density 150	Treat	Density	Interac
NH_4_ (mg/L)	2.97 ± 0.04^b^	1.97 ± 0.03^d^	2.37 ± 0.12^c^	3.87± 0.08^a^	0.67 ± 0.03^f^	0.84 ± 0.05^f^	1.31 ± 0.14^e^	<0.001	<0.001	<0.001
NH_3_ (mg/L)	0.18 ± 0.01^a^	0.06 ± 0.01^b^	0.015 ± 0.00^c^	0.01± 0.00^c^	0.04 ± 0.01^bc^	0.02 ± 0.00^bc^	0.02 ± 0.01^bc^	0.866	<0.001	0.315
NO_2_ (mg/L)	0.13 ± 0.00	30.33 ± 30.22	0.17 ± 0.00	0.19 ± 0.01	0.01 ± 0.00	0.03 ± 0.01	0.08 ± 0.01	0.32	0.412	0.41
NO_3_ (mg/L)	0.31 ± 0.01^cd^	0.28 ± 0.02^de^	0.24 ± 0.01^ef^	0.18 ± 0.02^f^	0.45 ± 0.02^a^	0.43 ± 0.01^ab^	0.3 ± 0.00^de^	<0.001	<0.001	0.001
ARRS^1^	0 ± 0^d^	33.47 ± 1.74^b^	20.32 ± 3.47^c^	-30.45 ± 1.79^e^	63 ± 0.25^a^	53.38 ± 2.33^a^	27.65 ± 5.74^bc^	<0.001	<0.001	<0.001
pH	8.05 ± 0.06^a^	7.68 ± 0.06^bc^	6.98 ± 0.07^ef^	6.73± 0.09^f^	7.95 ± 0.06^ab^	7.47 ± 0.07^cd^	7.32 ± 0.09^de^	<0.001	<0.001	0.048
O_2_ (mg/L)	5.89 ± 0.05^cd^	6.05 ± 0.07^bc^	5.52 ± 0.07^d^	4.8 ± 0.13^e^	6.75 ± 0.06^a^	6.31 ± 0.1^b^	5.89 ± 0.07^cd^	<0.001	<0.001	0.018

^1^Ammonia Removal Rate; as % of the Source (ARRS) = (TAN source–TAN treatment)

* 100/TAN source, where TAN is total ammonia nitrogen. Density, (fish/m^3^).

### 3.5 Efficiency in growth, survival, and food consumption

**Table 6** outlines the growth performance and survival rates of European seabass exposed to natural zeolite treatment and a control treatment without zeolite, across varying densities aimed at mitigating elevated ammonia concentrations. The results, with means and standard errors, reveal significant improvements in various growth parameters and survival rates in the zeolite-treated groups compared to the control. The final weight, weight gain, average daily gain (ADG), specific growth rate (SGR), and survival percentage consistently demonstrate substantial enhancements with zeolite application, particularly at higher fish densities. The hepatosomatic index (HSI) and condition factor (KF) also reflect positive trends with zeolite treatment. Statistical analysis indicates highly significant differences (*p < 0*.*001*) across treatments for most variables, emphasizing the positive impact of zeolite on the growth and health of European seabass under conditions of ammonia stress. The interaction effects between treatment and fish density further underscore the nuanced dynamics influencing the observed improvements. Overall, these findings highlight the potential of natural zeolite as a beneficial intervention for mitigating the negative effects of ammonia stress on the growth and survival of European seabass.

**Table 6:** Growth performance and survival rate of European seabass treated with natural zeolite as a removal of stressful ammonia concentrations.

Variable	Control			Zeolite			*P*-value		
	Density 50	Density 100	Density 150	Density 50	Density 100	Density 150	Treat	Density	Interac
Initial weight(g)	9.52 ± 0.22	8.64 ± 0.26	9.42 ± 0.37	8.6 ± 0.91	9.02 ± 0.60	9.36 ± 0.59	0.664	0.599	0.495
Final weight (g)	31.3 ± 0.29^b^	28.2 ± 1.2^c^	21.1 ± 0.4^d^	37.2 ± 0.75^a^	35.5 ± 0.38^a^	30.1 ± 0.54^bc^	<0.001	<0.001	0.086
Weight gain (g)	21.8 ± 0.23^b^	19.6 ± 1.15^b^	11.7 ± 0.1^c^	28.6 ± 1.46^a^	26.5 ± 0.69^a^	20.8 ± 0.324^b^	<0.001	<0.001	0.318
ADG (g/fish/d)	0.24 ± 0.00^b^	0.22 ± 0.01^b^	0.13 ± 0.00^c^	0.32 ± 0.02^a^	0.29 ± 0.01^a^	0.23 ± 0.00^b^	<0.001	<0.001	0.333
SGR (%/d)	0.58 ± 0.01^b^	0.57 ± 0.02^b^	0.39 ± 0.01^c^	0.71 ± 0.05^a^	0.66 ± 0.03^ab^	0.57 ± 0.02^b^	<0.001	<0.001	0.391
Survival rate (SR, %)	81.2 ± 1.18^b^	79.8 ± 1.11^b^	69.8 ± 3.59^c^	89.8 ± 0.85^a^	85.2 ± 1.25^ab^	82.5 ± 1.32^ab^	<0.001	<0.001	0.16
KF	1.07 ± 0.05^b^	0.9 ± 0.01^cd^	0.79 ± 0.01^d^	1.27 ± 0.03^a^	1.29 ± 0.03^a^	1.02 ± 0.03^bc^	<0.001	<0.001	0.008
HSI (%)	2.39 ± 0.06^a^	2.07 ± 0.04^b^	1.97 ± 0.03^b^	2.43 ± 0.04^a^	2.43 ± 0.02^a^	2.33 ± 0.04^a^	<0.001	<0.001	0.001

Density, (fish/m^3^); ADG, Average daily gain; SGR, Specific growth rate; KF, Condition factor; HSI, Hepatosomatic index

### 3.6 Feed utilization and carcass composition percentage of European seabass treated with natural zeolite

**Table 7** displays the feed utilization and proximal body analysis of juvenile European sea bass under natural zeolite treatment compared to a control treatment without zeolite, across different densities. The percentage body composition of juvenile *D*. *labrax* exhibited significant differences (*p* < 0.05) in all aspects except for body moisture. The result show that stocking density and zeolite had a significant (*p* < 0.05) impact on the feed conversion ratio (FCR) and the protein efficiency ratio (PER). The best results were seen when stocking density and zeolite were kept as low as possible. The findings clearly show that adding zeolite has a significant impact on the growth performance, feed utilization, and survival rate of juvenile seabass. The zeolite groups exhibited a statistically significant increase in final body protein levels (*p* < 0.05), although body ash and lipids were found to be lower compared to the control group. Additionally, research has shown that an increase in density causes a decrease in protein content as well as an increase in lipid and ash contents. The study observed the relationship between zeolite treatment and density, specifically in relation to ash. The results indicated that the maximum value of 18.8 ± 0.36% was seen in the high-density control group, while the lowest value of 11.6 ± 0.439% was observed in the low-density group with zeolite treatment.

**Table 7:** Feed utilization and carcass composition of European seabass treated with natural zeolite as removal of stressful ammonia concentrations.

Variable	Control			Zeolite			*P*-value		
	Density 50	Density 100	Density 150	Density 50	Density 100	Density 150	Treat	Density	Interac
**Feed utilization**									
Feed intake (g)	47.8 ± 1.6^a^	49.5 ± 2.79^a^	34.4 ± 0.95^b^	40.2 ± 3.36^ab^	46.1 ± 2.05^a^	41.7 ± 1.41^ab^	0.5	0.001	0.009
FCR	2.19 ± 0.08^c^	2.54 ± 0.04^b^	2.94 ± 0.1^a^	1.4 ± 0.06^e^	1.75 ± 0.09^d^	2.01 ± 0.06^cd^	<0.001	<0.001	0.538
PER	0.71 ± 0.03^cd^	0.61 ± 0.01^de^	0.53 ± 0.02^e^	1.11 ± 0.05^a^	0.89 ± 0.05^b^	0.77 ± 0.02^bc^	<0.001	<0.001	0.056
**Carcass composition (%)**									
Protein	49.7 ± 0.55^b^	44.3 ± 1.2^c^	40.4 ± 0.67^d^	57 ± 0.94^a^	52.5 ± 0.92^b^	50 ± 0.43^b^	<0.001	<0.001	0.367
Lipid	25.4 ± 0.32^bc^	28.1 ± 1.5^ab^	31.2 ± 1.03^a^	19.4 ± 1.29^d^	21.4 ± 1.01^cd^	22.4 ± 0.61^cd^	<0.001	0.002	0.387
Ash	13.1 ± 0.28^cd^	15.3 ± 0.54^b^	18.8 ± 0.36^a^	11.6 ± 0.44^d^	14.1 ± 0.12^bc^	15.6 ± 0.24^b^	<0.001	<0.001	0.027

Density, (fish/m^3^); FCR, Feed conversion ratio; PER, Protein efficiency ratio

### 3.7 Hematological analysis

**Table 8** displays the hematological parameters of European seabass under natural zeolite treatment and a control treatment without zeolite, across various fish densities, for mitigating stressful ammonia concentrations. The data, expressed as means with standard errors, reveal significant improvements in several hematological indicators in the zeolite-treated groups compared to the control. Zeolite application, particularly at higher fish densities, leads to a significant increase in red blood cell count (RBCs) and hemoglobin levels (Hb), emphasizing the positive impact on oxygen-carrying capacity. Hematocrit (Hct) also exhibits improvements with zeolite treatment. White blood cell count (WBCs) and differential leukocyte percentages (lymphocytes, monocytes, neutrophils) indicate a positive influence on immune response, with higher fish densities showing enhanced immune parameters. Statistical analysis demonstrates highly significant differences *(p < 0*.*001*) across treatments for most variables, highlighting the positive effects of zeolite in ameliorating hematological stress induced by ammonia. The interaction effects between treatment and fish density provide valuable insights into the nuanced dynamics influencing these hematological responses. Overall, the results suggest that natural zeolite treatment contributes to improved hematological health in European seabass exposed to ammonia stress.

**Table 8:** Hematological parameters of European seabass treated with natural zeolite as removal of stressful ammonia concentrations.

Variable	Control			Zeolite			*P*-value		
	Density 50	Density 100	Density 150	Density 50	Density 100	Density 150	Treat	Density	Interac
RBCs (× 10^6^/mm^3^)	1.38 ± 0.06^b^	1.30 ± 0.05^b^	1.15 ± 0.05^b^	2.16 ± 0.19^a^	1.92 ± 0.14^a^	1.66 ± 0.12^ab^	<0.001	0.018	0.521
Hb (g/dl)	6.36 ± 0.07^c^	6.38 ± 0.16^c^	6.06 ± 0.11^c^	9.35 ± 0.20^a^	8.77 ± 0.24^a^	7.85 ± 0.22^b^	<0.001	<0.001	0.012
Hct (%)	16.75 ± 0.27^b^	16.42 ± 0.24^b^	15.86 ± 0.23^b^	28.71 ± 2.51^a^	28.02 ± 2.66^a^	25.34 ± 2.89^a^	<0.001	0.52	0.784
WBCs (10^3^/mm^3^)	21.74 ± 0.33^b^	20.9 ± 0.28^b^	21.18 ± 0.43^b^	33.05 ± 1.77^a^	30.72 ± 2.60^a^	29.44 ± 1.92^a^	<0.001	0.378	0.614
Lymphocytes (%)	54.64 ± 4.55^bc^	53.34 ± 4.24^c^	52 ± 3.59^c^	71.91 ± 0.68^a^	69.56 ± 1.611^a^	66.44 ± 1.363^ab^	<0.001	0.432	0.898
Monocytes (%)	2.22 ± 0.06^cd^	2.16 ± 0.03^cd^	1.99 ± 0.05^d^	2.64 ± 0.0675^a^	2.52 ± 0.09^ab^	2.34 ± 0.07^bc^	<0.001	0.002	0.824
Neutrophils (%)	16.77 ± 0.38^b^	16.42 ± 0.27^b^	15.88 ± 0.21^b^	21.94 ± 0.39^a^	21.38 ± 0.65^a^	20.19 ± 0.56^a^	<0.001	0.023	0.599

Density, (fish/m^3^); RBC, Red blood cell count; Hb, Hemoglobin; HCT, Hematocrit; WBC, White blood cell count.

### 3.8 Serum biochemical values

**Table 9** presents the biochemical parameters of European seabass under natural zeolite treatment and a control treatment without zeolite, across various fish densities, for mitigating stressful ammonia concentrations. The results, with means and standard errors, highlight significant improvements in multiple biochemical markers in zeolite-treated groups compared to the control. Notably, cholesterol levels exhibit a dose-dependent decrease with increasing fish density, emphasizing the positive impact of zeolite. The total protein and albumin levels, while showing some variations, do not reach statistical significance. Glucose levels are significantly elevated in zeolite-treated groups, suggesting a potential metabolic response to zeolite application. The albumin/globulin ratio (A/G) significantly increases with zeolite treatment, indicative of improved protein metabolism. Urea, uric acid, and lysozyme levels demonstrate mixed trends across treatments. Importantly, zeolite treatment leads to significant reductions in aspartate aminotransferase (AST) and alanine aminotransferase (ALT), indicating a protective effect on liver function. Statistical analysis underscores the significance of zeolite treatment for most parameters (*p < 0*.*001*), with notable interactions between treatment and fish density. These findings suggest that natural zeolite application contributes to favorable biochemical profiles, showcasing its potential in mitigating the biochemical impacts of ammonia stress in European seabass.

**Table 9:** Biochemical parameters of European seabass treated with natural zeolite as removal of stressful ammonia concentrations.

Variable	Control			Zeolite			*P*-value		
	Density 50	Density 100	Density 150	Density 50	Density 100	Density 150	Treat	Density	Interac
Cholesterol (mg/dl)	244.8 ± 19.52^b^	267.8 ± 8.09^ab^	325.7 ± 22.59^a^	178.5 ± 6.04^c^	209.8 ± 3.09^bc^	222.8 ± 4.59^bc^	<0.001	0.001	0.215
T- protein (g/dL)	4.04 ± 0.29	3.88 ± 0.16	4.21 ± 0.09	5.54 ± 1.14	5.01 ± 1.03	4.06 ± 0.64	0.085	0.619	0.472
Albumin (g/dL)	1.47 ± 0.13^ac^	1.73 ± 0.10^ab^	1.84 ± 0.08^a^	1.24 ± 0.12^bc^	1.13 ± 0.21^c^	0.92 ± 0.12^c^	<0.001	0.827	0.057
Glucose (mg/ dl)	2.57 ± 0.16	2.15 ± 0.18	2.37 ± 0.07	4.31 ± 1.19	3.88 ± 1.03	3.14 ± 0.535	0.021	0.609	0.723
A/ G	0.57 ± 0.02^ac^	0.83 ± 0.12^a^	0.78 ± 0.04^ab^	0.38 ± 0.12^bc^	0.37 ± 0.14^bc^	0.30 ± 0.02^c^	<0.001	0.431	0.23
Urea (mg dl)	43.5 ± 2.72	42.25 ± 3.33	46.5 ± 3.57	37.5 ± 0.96	38.5 ± 1.19	42 ± 1.23	0.027	0.218	0.894
Uric Acid (mg/dl)	3.13 ± 0.44	3.04± 0.56	3.8 ± 0.57	2.38 ± 0.17	2.29 ± 0.18	2.55 ± 0.18	0.01	0.396	0.767
Lysozyme (mg/dL)	0.32 ± 0.01^c^	0.26 ± 0.00^cd^	0.2 ± 0.01^d^	0.55 ± 0.02^a^	0.52 ± 0.02^ab^	0.46 ± 0.02^b^	<0.001	<0.001	0.401
AST (IU/L)	37.04 ± 0.07^c^	42.12 ± 0.15^b^	46.33 ± 0.4^a^	25.72 ± 0.28^e^	33.14 ± 0.33^d^	35.99 ± 0.10^c^	<0.001	<0.001	0.001
ALT (IU/L)	36.02 ± 0.1^b^	44.94 ± 0.13^a^	49.7 ± 0.28^a^	26.8 ± 2.563^c^	24.78 ± 2.72^c^	36.28 ± 0.48^b^	<0.001	<0.001	0.008

Density, (fish/m^3^); AST, Aspartate aminotransferase; ALT, Alanine aminotransferase

## 4. Discussion

There were two main goals of this study: to find out if natural zeolite could help with two big environmental problems that aquaculture faces: getting rid of heavy metals and reducing ammonia stress in young European sea bass. This investigation specifically targeted underground water sources with varying densities. Naturally-occurring substances known as heavy metals may pose significant threats to both aquatic ecosystems and human health when they are found in water in excessive amounts [61]. In the present study, we observed the mean values of some heavy metals’ values between control and zeolite groups (cadmium, copper, zinc iron, and lead) in water at different densities. It was found that the zeolite group had lower concentrations of Cd, Cu, Zn, and Fe compared to the control group. This happened at different densities. Consistent with our results, studies have shown that natural zeolites can remove heavy metal cations (Mn, Fe, Cd, Zn, Pb, Cu, Co, Cr, Cu, and Pb) from waste and drinking water up to 97% of the time [33].

The potential mechanisms through which zeolite contributes to the removal of heavy metals involve various factors, primarily centered around its exceptional adsorption capacities and interactions with metal ions [62]. Zeolites, owing to their unique crystal structures and high surface areas, possess inherent adsorption sites that attract and retain heavy metal ions. The ion exchange capability of zeolites allows them to selectively replace cations in their structure with heavy metal ions, facilitating the removal of these contaminants from aqueous solutions [63]. Additionally, the surface chemistry of zeolites plays a crucial role in metal adsorption, with active functional groups enhancing binding affinity. Moreover, the specific mineralogical composition of zeolites influences their metal sorption capacities [64]. Previous studies, such as those by Figueiredo and Quintelas [65] and Roshanfekr Rad and Anbia [66], have extensively explored and supported these mechanisms, providing valuable insights into the intricate processes by which zeolites effectively contribute to the removal of heavy metals from various environmental matrices.

Additionally, earlier research has shown zeolite’s capacity to absorb heavy metals from wastewater from industry, municipalities, and agriculture [67]. During the course of the experiment, it was observed that the zeolite group with a density of 150 had depressed levels of Cd and Pb. Consistent with our research findings, previous reports have shown that the inclusion of zeolite in water had a beneficial impact on the elimination of Cd and Pb contaminants from *Oreochromis mossambicus* [68]. One potential factor contributing to our findings might be the insufficient duration of the study period, which may have hindered the total elimination of Cd and Pb from seabass. Additionally, the density of the seabass population could have had a role in these outcomes. Our findings also showed that the percentage of heavy metal removal (RRS%) from water increased with density. Additionally, the RRS% of Cd, Cu, Zn, and Fe was greatly boosted by the addition of zeolite. Accordingly, when zeolite treatment is applied, the percentage of ammonia removal rate at varying densities increases considerably for all heavy metals (Cd, Cu, Zn, Fe, and Pb) when compared to the control group. Erdem et al. suggested that natural zeolites had significant capabilities for the removal of heavy metals [69].

Our results showed that the addition of zeolite has a substantial and favorable influence on lowering heavy metal accumulation in the liver, gills, and muscles of sea bass across all densities. Furthermore, increasing fish density is related to lower heavy metal accumulations in all organs of sea bass. In agreement with our findings, James and Sampath discovered that the presence of zeolite in water reduced heavy metals such as Cd in the muscle of *Oreochromis mossambicus* [68]. Similarly, the zeolite-treated group had the lowest accumulation of heavy metals such as Cu in the liver and muscle of *Oreochromis niloticus* [70]. The results also showed that the zeolite treatment made the water quality better by lowering the levels of NH_4_, NH_3_, and NO_2_ by a lot compared to the control treatments. This led to higher readings for DO, pH, and ammonia. Furthermore, there was a significant relationship between stocking density (SD) and zeolite treatment for all water quality metrics. Saeed et al. came to the same conclusion, showing that adding zeolite greatly decreased the amounts of all inorganic dissolved nitrogen in water, including ammonia (NH_3_, NH_4_-N), nitrite (NO_2_), and nitrate (NO_3_) [71]. This is consistent with our findings, since NO_2_ levels fall dramatically as the zeolite level increases. This discovery might be attributed mostly to zeolite’s adsorption ability, which attracted nitrate ions and enhanced nutrient preservation from water [72]. In addition, we noticed an increase in water pH and DO in the treatment group as compared to the control group. Finding high pH levels might have something to do with the fact that zeolites are slightly acidic and sodium-form exchangers prefer hydrogen. This means that high pH levels are found when the exchanger is balanced with electrolyte solutions that aren’t very strong [73].

The incorporation of zeolite into the water or feed used in fish raising has several advantages that have both direct and indirect impacts on the well-being of aquatic organisms. The current investigation documented an enhancement in growth and feed utilization indices (namely, FBW, WG, ADG, SGR%, survival, and KF) of European sea bass as the concentration of zeolite increased. Similar to this study, Ali et al. discovered that adding natural zeolite to tanks for rearing as a water adjuvant increased the ADG and survival rate of *Dicentrarchus labrax* [12]. Similarly, Rabiatul et al. and Aly et al. found a significant positive correlation between the development efficacy of *Oreochromis sp*. and *D*. *labrax*, and increasing zeolite levels [16, 74]. The feed utilization and carcass composition of European seabass treated with natural zeolite demonstrated that except for body moisture, there were significant variations in the body composition of juvenile *D*. *labrax* (*p* < 0.05). According to the findings, stocking density and zeolite had a significant (*p* < 0.05) influence on FCR and PER. Recently it has been reported that stocking density had a negative effect, but PER and FCR got much better when the zeolite level went up. However, a substantial rise in body protein was seen, and when zeolite was increased, the negative effects of increased density were lessened. Meanwhile, the quantity of fat and ash decreased [39]. The enhancement of fish growth performance and feed utilization by zeolite can be attributed to its effectiveness in reducing ammonia levels and toxicity, thereby improving water quality. This improvement in water conditions enables the conservation of energy that would otherwise be expended in coping with adverse environmental factors, redirecting it towards growth [75, 76].

The hematological parameters of the fish mirrored the escalating deterioration of the surrounding rearing water, and these measurements can serve as valuable tools for regular assessments of the physiological status of the fish [77, 78]. The current result demonstrated that the incorporation of zeolite had a favorable and advantageous impact on crucial hematological parameters in juvenile *D*. *labrax* (European seabass) in comparison to a control group. There are many things that can change the blood parameters of fish, including the season, the quality of the water, the fish’s age, its sex, its nutrition, its health, its genetic makeup, how it was transported and handled, and other environmental conditions, as well as the ways it was sampled and analyzed in the lab [79, 80]. Biochemical parameters are commonly employed for assessing the physiological and overall health condition of aquatic organisms [81–83]. Elevated ALT and AST activities are acknowledged as markers reflecting the liver’s health status [84–86]. The result of the present study significantly increases the albumin, cholesterol, and urea, as well as liver function enzymes such as AST and ALT, in the European sea bass exposed to the highest density. There are compensatory effects of zeolite on the levels of total protein and globulin at the maximal density of fish rearing. Furthermore, the water interventions resulted in considerable alleviation of cholesterol, urea, AST, and ALT levels in both densities. Similarly, Çoğun and Şahin observed that the administration of zeolite effectively mitigated the elevated levels of serum cortisol, ALT, AST, and cholinesterase in Nile tilapia subjected to lead-induced poisoning [87].

## 5. Conclusions

The study demonstrates that incorporating zeolite into the water significantly enhances water quality metrics, effectively mitigating the adverse effects of increased aquaculture density on water quality indicators. Elevated stocking densities of European sea bass resulted in a noticeable decline in growth performance, feed utilization, and various hemato-biochemical indices. However, upon introducing zeolite, improvements were observed in feed utilization, hematological parameters, and biochemical aspects of fish growth performance. The study’s findings highlight the efficacy of zeolite (15 ppt) in reducing heavy metal deposition in both water and fish organs, contributing to enhanced fish growth and development. Despite an increase in density, the study reveals a substantial reduction in the buildup of heavy metals in water and fish organs. This research underscores the potential of natural zeolites to alleviate the impact of water quality issues, showcasing their effectiveness in diminishing heavy metal deposition in fish tissues by reducing their availability in polluted water.

## Supporting information

S1 Data (XLSX)

## Abbreviations

SD: Stocking density
DM: Dry matter
DO_2_: Dissolved oxygen
TAN: Total ammonia nitrogen
ARRS: Ammonia Removal Rate; as % of the Source
ARRC: Ammonia Removal Rate; as % of the Control
ADG: Average daily gain
SGR: Specific growth rate
KF: Condition factor
HSI: Hepatosomatic index
FCR: Feed conversion ratio
PER: Protein efficiency ratio
RBC: Red blood cell count
Hb: Hemoglobin
HCT: Hematocrit
WBC: White blood cell count
AST: Aspartate aminotransferase
ALT: Alanine aminotransferase
